# Supplementary material for: Trajectory simulation of multi-body parachute system for airdrop-capable UAVs based on fluid-structure interaction
Source: PLoS One. 2026 Mar 5;21(3):e0343305. doi: 10.1371/journal.pone.0343305 (PMC12962541; doi:10.1371/journal.pone.0343305)
Supplement: Attachment — Submitted filename: Response to Reviewers.docx [file pone.0343305.s002.docx]

Dear reviewers,

Thank you very much for your comments and professional advice on our manuscript entitled “Trajectory Simulation of Multi-body Parachute System for Airdrop-capable UAVs Based on Fluid-Structure Interaction” (PONE-D-25-45360R1). These opinions help to improve academic rigor of our article. Based on your suggestion and request, we have made corrected modifications on the revised manuscript. We hope that our work can be improved again. Furthermore, we would like to show the details as follows:

**Reviewer #1，**

1. Although the paper employs complex cross-validation of simulations, all results stem from "simulation-to-simulation" comparisons, lacking final verification against real-world physical experimental data (e.g., high-speed photogrammetric trajectory measurements, sensor-based attitude/velocity data). In engineering research, simulation models must be validated against experimental data to verify their boundary conditions and actual accuracy; otherwise, their effectiveness in real-world environments cannot be proven .

**Response 1：**

We fully agree with your point: relying solely on cross-validation through "simulation-to-simulation" comparisons is insufficient to demonstrate the model’s effectiveness in real-world engineering environments. In the revised manuscript, we have added a section on validation through data from field airdrop experiments. We conducted physical airdrop tests using a 1:1 prototype of the drone and its parachute. Real-time attitude angles and velocity data during descent were recorded using onboard high-precision IMU and GPS sensors. We have also included an on-site photo of the experiment (Fig. 12) and comparative data curves (Fig. 13). The results show that the relative error of the trajectory is controlled within 8%, which fully verifies the accuracy and robustness of the model in real-world conditions.

2. The paper overlooks key environmental factors (e.g., external wind field disturbances, nonlinear aerodynamic effects) and fails to quantify the impact degree of these simplifications through sensitivity analysis. For instance, ignoring wind fields under complex atmospheric turbulence could lead to trajectory prediction deviations exceeding 20%, but the authors do not discuss the range of such errors, undermining the model's practical applicability .

**Response 2：**

We sincerely appreciate the reviewer’s insights. We fully agree that external disturbances and nonlinear aerodynamic forces are critical for the system’s practical engineering applications. In the revised manuscript, we have explicitly acknowledged this limitation. However, we kindly ask the reviewer to understand that conducting a comprehensive sensitivity analysis of environmental factors is beyond the scope of this paper, for the following reasons: The primary objective of this study is to establish and validate a rigid-flexible coupling mechanism based on Kane’s method. We aim to demonstrate that the mathematical model can accurately capture the dynamic interactions between the UAV and the parachute under ideal conditions. Therefore, this work is positioned as a theoretical benchmark. As noted in the revised conclusion and discussion sections, we have explicitly included external wind field perturbations as the next step in our research roadmap.

3. The method for obtaining aerodynamic coefficients (e.g., lift/drag coefficients) in the multi-body dynamics model is not detailed, merely mentioned as "based on CFD data" without specifying the calibration process or citing sources. Furthermore, the absence of key parameters in the FSI simulation, such as grid convergence analysis and time step settings, prevents other researchers from replicating the experiment .

**Response 3：**

We thank the reviewer for pointing out these issues. In response, we have made the following improvements in the revised manuscript, which are highlighted in yellow:

1. The lift and drag coefficients of the parachute and the main body after parachute deployment are now based on CFD simulations. We have applied the least squares method to fit these into an aerodynamic database construction approach.
2. We have added a grid independence verification. The results from three different mesh configurations were compared, which validates the mesh scale selected in the study. In the FSI setup section, we have specified the time step settings: a time step of 0.01 s, with a maximum of 30 coupling iterations per time step. To accommodate the large deformations of the parachute, a diffusion-based smoothing method was applied to update the fluid domain mesh, and the convergence criterion was set to an RMS residual of 10^-4^. The corresponding changes have been highlighted in yellow in the manuscript.
4. The literature review merely lists existing methods without critically pointing out their limitations (e.g., most models do not couple time-varying wind fields with multi-temperature zone cooperative optimization). An analysis of the research gap should be added to clarify this paper's innovation points .

**Response 4：**

We sincerely thank the reviewer for this highly constructive suggestion. Following your advice, we have rewritten the literature review summary paragraph, adding a critical analysis, clarifying the research gaps, and highlighting the contributions of this paper. The specific revisions are marked in yellow in the introduction section of the revised manuscript.

5. The calibration method for aerodynamic coefficients is ambiguous, stated only as "based on CFD data" without specifying the specific conditions (e.g., angle of attack range, Reynolds number). It is suggested to present comparative errors between calibration data and experimental values in a table.

**Response 5：**

Thank you for the reviewer's comments. We agree that specifying the CFD calculation conditions is crucial for the reproducibility of the study. In the revised manuscript, we have supplemented the specific operational range: the angle of attack ranges from 90°±30°, and the initial velocity ranges from 0-50 m/s. Given the difficulty in obtaining static wind tunnel data for the flexible parachute canopy, the accuracy of these coefficients was validated through comparisons with actual flight trajectories. As shown in Figure 13 and Table 4, the relative errors between the key trajectory parameters derived from the simulation and the actual airdrop test data are less than 8%.

6. Comparisons with baseline algorithms are mentioned as "superior" without presenting specific data (e.g., convergence iteration count, CPU time). Quantitative metrics need to be supplemented .

**Response 6：**

We thank the reviewer for the suggestion. We fully agree that quantitative data is required to objectively demonstrate the advantages of the method presented in this paper. We conducted a comparative study with a standard Rigid Body Dynamics (RBD) benchmark, as summarized in Table 3. The results show that although the RBD benchmark is low in computational cost, its trajectory deviation can be as high as 46.67%. In contrast, the Kane-based rigid-flexible coupling model proposed in this paper significantly reduces the prediction error. While the CPU time increases by approximately 30%, it still fully meets the requirements for real-time applications compared to the several hours required for FSI simulations. Therefore, the method presented in this paper is considered superior, as it effectively eliminates the large deviations inherent in the benchmark algorithm while maintaining high efficiency.

**Reviewer #2，**

1. Check the grammar of the entire manuscript. It needs to be further made more accurate to improve readability.

**Response 1：**

We sincerely thank the reviewer for their attention to the linguistic quality of the paper. We greatly value this feedback and have corrected the grammatical errors, spelling mistakes, and inappropriate punctuation throughout the text. Additionally, we have rephrased certain long and complex sentences as well as awkwardly worded paragraphs to improve logical flow, readability, and ensure the precision of the academic expression.

2. Introduction, “Current mainstream air transport methods... low transport efficiency [1].”…….., This paragraph only points out the shortcomings of traditional air transport methods but fails to clarify the specific deficiencies of existing airdrop UAVs in parachute-UAV coupling modeling (e.g., traditional models ignore the impact of parachute flexible deformation on trajectory, insufficient integration of FSI simulation and multibody dynamics). It is recommended to supplement the direct correlation between existing research gaps and the work of this paper to strengthen the research motivation.

**Response 2：**

We fully agree with your viewpoint that merely presenting a macro-level comparison is insufficient to adequately support the research motivation regarding the "dynamic modeling" aspect of this study. We have rewritten and expanded the introduction, supplementing it with the specific research gaps identified in existing studies and their direct relevance to the work presented in this paper, thereby strengthening the research motivation. The specific revisions are indicated in the manuscript with yellow highlighting.

3. Research object, “Control is exclusively implemented via two control surfaces positioned at the left and right trailing edges of the rear wing.”Key parameters of the control surfaces (e.g., deflection angle range, response time, control efficiency coefficient) are not explained. These parameters are crucial for the attitude control of the UAV after separation. It is recommended to supplement relevant parameters to improve the model description of the research object.

**Response 3：**

We sincerely thank the reviewer for this detailed and professional suggestion. It is indeed important to enhance the description of the control surface parameters for the UAV. In response, we have added parameters of the control surfaces in the manuscript, including maximum deflection angle and servo response time, with the additions highlighted in yellow. We would like to take this opportunity to clarify that the focus of this study is on the airdrop descent trajectory of the "parachute-UAV system." At this stage, the UAV's control surfaces are not utilized for active attitude control. These parameters are critical for the subsequent "pull-up and cruise" phase after separation, which constitutes the core focus of our next-phase research.

4. Table 1 suggests consistent units, mm/m

**Response 4：**

We thank the reviewer for the suggestion. We have unified all geometric parameters in Table 1 to meters (m). The corresponding values have been reconverted and verified.

5. FSI simulation analysis, “The computational fluid domain measuring 80 m (L) × 30 m (W) × 150 m (H) is illustrated in Fig 5.”The basis for selecting the computational fluid domain size (80m×30m×150m) is not explained, such as whether the maximum deployment size of the parachute and flow field boundary effects (avoiding wall interference) are considered. It is recommended to supplement the rationality analysis for size determination

**Response 5：**

We would like to thank the reviewer for raising this point. When setting the computational domain dimensions (80 * 30 * 150 meters), under the condition of the fully deployed parachute with its maximum diameter, the ratio of the parachute’s projected area to the cross-sectional area of the computational domain is only 0.47%, which is well below the commonly recommended threshold of 3%. The distance from the center of the parachute to the nearest side wall is 15 meters, which is 15 times the characteristic diameter, sufficient to eliminate wall effects. The vertical height is set to 150 meters, which not only covers the maximum vertical stabilization distance (approximately 120 meters) but also provides a downstream region exceeding 20 times the diameter to adequately capture the development of wake vortices. The relevant explanations have been highlighted in yellow in the manuscript.

6. Multibody dynamics model validation, “Validation data confirms that both simulation methods achieve coordinate errors within ±5%”, The statistical type of error (e.g., Root Mean Square Error RMSE, Maximum Absolute Error MAE, average relative error) and sample size (e.g., statistical results of how many time nodes or spatial coordinate points) are not clarified. It is recommended to supplement the specific method and statistical details of error calculation to improve result credibility.

**Response 6：**

We thank the reviewer for the suggestion. We fully agree that quantitative comparative data are needed to support the conclusions on accuracy presented in the paper. In the revised manuscript, we have added Table 3. This table details the relative errors between the dynamic model proposed in this paper and the high-fidelity FSI simulation results in terms of horizontal displacement and vertical displacement. Comparative data indicate that the conclusion of ≤5% mentioned in the text holds true for the vast majority of working conditions.

7. Airdrop trajectory deviation analysis, “under extreme operating conditions characterized by initial velocities ≥45 m/s and parachute diameters <0.8 m”, The basis for determining "extreme operating conditions" is not defined (e.g., load limits based on engineering application scenarios, operating condition classification standards in similar studies in the literature, or critical conditions where trajectory deviation increases significantly in simulations). It is recommended to supplement the definition basis to make the operating condition classification more reasonable.

**Response 7：**

We thank the reviewer for the suggestion. The definition of extreme operating conditions in the paper primarily references the rapid deployment mission requirements and application scenarios. In engineering applications, to improve payload survivability and minimize flow disturbances, the initial deployment velocity is often set near the upper limit of the system design envelope (approximately 45 m/s). Additionally, this definition is based on critical phenomena observed in simulation experiments. When the initial velocity reaches 45 m/s and the parachute diameter is reduced below 0.8 m, the aerodynamic instability effects of the system significantly intensify, and the ballistic deviation exhibits a nonlinear growth trend. We have supplemented the relevant engineering context in the revised manuscript and highlighted it in yellow.

8. Parachute jettison points selection, “Limiting the horizontal velocity component (Vₓ<3 m/s) mitigates post-separation nonlinear effects”, The reason for selecting "3m/s" as the horizontal velocity threshold is not explained. It is recommended to supplement the simulation or experimental basis for threshold determination to enhance the persuasiveness of the conclusion.

**Response 8：**

We thank the reviewer for the suggestion. The setting of the horizontal velocity threshold during parachute cutoff is primarily constrained by the following engineering considerations. First, the requirement for landing accuracy: if the horizontal velocity exceeds this threshold when descending from the mission altitude, the horizontal displacement caused by inertia will lead to a landing point deviation beyond the permissible engineering tolerance range. Second, the constraint of separation safety: a lower horizontal velocity significantly reduces the risk of secondary collisions between the parachute canopy and the payload after cutoff, thereby decreasing the pressure on the subsequent pull-up maneuver. Considering the Monte Carlo simulation results shown in Figure 15 and the engineering constraints, this value (3 m/s) aligns with the mission design standards of existing airdrop recovery systems, effectively balancing mission success rate and system dynamic stability. The relevant rationale has been added and highlighted in yellow in the manuscript.

9. Equation 4, The specific definitions of "S" (reference area) and "L" (characteristic length) in the formula are not clarified (e.g., whether S is the parachute projected area or UAV windward area, and L is the parachute diameter or wingspan). It is recommended to supplement the complete parameter definitions to ensure the formula is reproducible.

**Response 9：**

We thank the reviewer for pointing out this omission. Parameter definitions are crucial for the reproducibility of the study. In the revised manuscript, we have supplemented and clarified the parameter description following Equation 4. In this study, S is defined as the projected area of the parachute, and L is defined as the diameter of the parachute.

10. FSI simulation analysis, “The mesh employs hexahedral-dominant topology... inflation-layer generation technique.”Key parameters of the inflation layer (e.g., number of layers, growth rate, ratio of the first layer grid height to wall distance y⁺) are not explained. These parameters directly affect the calculation accuracy of the near-wall flow field. It is recommended to supplement detailed parameters of mesh generation to improve simulation reproducibility.

**Response 10：**

We thank the reviewer for the suggestion. In the revised manuscript, we have supplemented detailed grid parameters. Specifically, to accurately capture the boundary layer effects, we set the height of the first grid layer to 0.05 mm, with a growth rate of 1.2, totaling 23 layers. The dimensionless wall distance y+ is approximately 10, verifying the reasonableness of the grid strategy.

11. FSI simulation analysis, “High-resolution elements (size: 0.001 m) applied at wing surfaces and parachute riser connection points”, The grid size of 0.001m is too fine, which may lead to excessively high computational cost. It is recommended to supplement the grid independence verification results (e.g., compare the calculation results of 0.001m, 0.002m, and 0.005m grids to prove that the 0.001m grid meets the accuracy requirements and has no significant error reduction) to explain the rationality of grid size selection.

**Response 11：**

We thank the reviewer for pointing out this issue. We have added content on grid independence verification. The computational results from coarse, medium, and fine grids were compared, demonstrating that the "medium-scale grid" selected in the paper achieves a balance between computational accuracy and efficiency . In the FSI setup section, we have detailed the coupled time step settings. Despite the higher computational cost associated with the chosen grid, it was ultimately selected to accurately capture the complex flow separation at the parachute-cable connection points and to maintain a reasonable distribution. The relevant modifications have been highlighted in yellow in the manuscript.

12. Flexible deformation induces variations, “the modal shape coefficient η characterizes the proportional contribution of deformation to the angle of attack”, The value range or determination method of the modal shape coefficient η is not explained. The rationality of η directly affects the accuracy of the rigid-flexible coupling model. It is recommended to supplement the determination method and verification results of η.

**Response 12：**

We thank the reviewer for pointing out this issue. η is a coupling coefficient introduced to characterize the influence of parachute canopy "breathing" and deformation on the effective angle of attack. As noted in the literature by Doherr et al., the aerodynamic forces of flexible parachute canopies exhibit unsteady and stochastic characteristics. Therefore, when establishing the system-level dynamic model, adopting a typical η value to represent the moderate stiffness coupling scenario is a standard approach, which aligns with the dynamic characteristics described in the literature.

**Reviewer #3，**

1. Although the introduction reviews numerous relevant studies, the discussion mostly remains at the level of "who did what research". Please conduct an in-depth comparison of the similarities and differences between existing studies and this work, and state the core contributions and novelty more clearly in the introduction and conclusion.

**Response 1：**

We sincerely thank the reviewer for this suggestion. In accordance with your advice, we have rewritten the literature review summary paragraph in the introduction section, adding critical analysis, clarifying the research gaps, and highlighting the contributions of this paper. The specific revisions are marked in yellow in the introduction section.

2. For the conclusion section, it would be better to adding a discussion on the research limitations and future research.

**Response 2：**

We thank the reviewer for the suggestion. We fully agree that discussing the limitations of the study and future directions plays a crucial role in enhancing the rigor of the paper. We have objectively pointed out the shortcomings of this study in terms of model simplification and environmental modeling, and proposed corresponding future research directions based on these. The specific revisions have been highlighted in yellow in the manuscript.

3. The selection of key parameters should be specifically explained.For example, what are the physical meaning and numerical source of the shape coefficients η (Equation (2))? The aerodynamic coupling coefficients k (Equations (3)-(4)) linearly correlate structural deformation with changes in aerodynamic forces/moments. How are they determined? And so on.

**Response 3：**

We thank the reviewer for pointing out this issue. η is a coupling coefficient introduced to characterize the effect of canopy "breathing" and deformation on the effective angle of attack. As noted in the literature by Doherr et al., the aerodynamic forces on flexible canopies exhibit unsteady and stochastic behavior. Therefore, in establishing a system-level dynamic model, using a typical η value to represent a moderate stiffness coupling scenario is a common practice. The coupling coefficients $k_{D,i}$ and $k_{L,i}$ are determined through parameter identification based on CFD simulation results. We calculated the aerodynamic forces under different modal deformation amplitudes and used the least squares method to fit a linear relationship between the generalized velocities and the variations in aerodynamic forces. $k_{D,i}$ quantifies the additional drag caused by modal shape variations, while $k_{L,i}$ represents the aerodynamic damping effect associated with modal velocity, which affects lift.

4. Please ensure that all variables used are defined when first introduced.

**Response 4：**

We thank the reviewer for the careful correction. We have thoroughly proofread the entire manuscript to ensure that every mathematical symbol and variable is clearly defined upon its first occurrence in the text.

5. The authors validate the multibody dynamics model via FSI simulation. Could it also be considered to include a rigid-body model for comparative analysis, so as to highlight the effectiveness of the research in this paper?

**Response 5：**

We thank the reviewer for the constructive suggestion. Introducing a rigid-body model as a benchmark is indeed necessary to demonstrate the importance of considering structural flexibility. We have added a comparative analysis involving a rigid-body model (RBD). The results, presented in the newly added Table 3, show that while the rigid-body model captures the general descent trend, it exhibits significant errors due to its neglect of aeroelastic effects. In contrast, the flexible model proposed in this paper shows good agreement with the FSI results.

6. Section "Parachute jettison points selection": Add a short discussion to illustrate the applicable scope or potential constraints of the obtained criteria.

**Response 6：**

We thank the reviewer for the suggestion. We fully agree on the necessity to clarify the scope of application for these selection criteria. It is explicitly noted that, while the qualitative principles are universal, the specific quantitative thresholds are customized based on the aerodynamic characteristics and structural stiffness of the UAV in this study. Therefore, for UAVs with different mass distributions or aerodynamic layouts, these thresholds should be recalibrated according to their respective flight envelopes. Additionally, under different meteorological conditions, varying convergence criteria are required to ensure safety. The specific revisions have been highlighted in yellow in the manuscript.

**Reviewer #4，**

1. Section Multi-body dynamics (Kane’s method) adopts several simplifying assumptions (constant gravity, neglecting crosswinds, small post-inflation deformation). These assumptions may significantly influence trajectory accuracy. A brief justification and discussion of their expected impact should be added.

**Response 1：**

We sincerely thank the reviewer for the rigorous review of the model assumptions. In the revised manuscript, we have added a discussion on the reasonableness of these assumptions, highlighted in yellow. Given that the descent altitude for the mission is approximately 500 meters, the variation in gravitational acceleration is less than 0.04%, which is negligible. This study focuses on the inherent stability of the rigid-flexible coupled system, and environmental factors will be considered in future robust control research. As demonstrated in the comparative validation with the FSI model in Table 3, the maximum trajectory error between the simplified model and the high-fidelity FSI model is controlled within 5%. This confirms that during the stable descent phase, the "small deformation" assumption is sufficient to capture the dominant global dynamic characteristics.

2. Equation (1) introduces a 6-mode vibration model, but the selection of modal shapes, natural frequencies, and damping ratios is not explained. Provide sources or reasoning for these parameters.

**Response 2：**

We thank the reviewer for pointing this out. We have clarified and annotated the sources of these parameters: The natural frequencies $\omega_{n1}$ in $K=\text{diag}\left( \omega_{n1}^{2},\ldots,\omega_{n6}^{2} \right)$ are obtained from a preliminary finite element modal analysis of the canopy in its inflated state. The first six dominant modes (breathing, swinging, and torsional modes) are selected to construct the model. As per the definition $C=\text{diag}\left( 2\zeta_{1}\omega_{n1},\ldots,2\zeta_{6}\omega_{n6} \right)$, the damping is dependent on the damping ratios. Since fabric damping is difficult to measure directly during flight, a standard empirical structural damping ratio, based on experimental data for nylon parachute canopies from the referenced literature, is adopted.

3. In Eq. (3) and Eq. (4), the coupling coefficients are introduced without describing how they were determined (empirical, from CFD, or fitted). Please specify their origin and role in the model.

**Response 3：**

We thank the reviewer for the correction. We fully agree that clarifying the method for determining the coupling coefficients is crucial. The coupling coefficients $k_{D,i}$ and $k_{L,i}$ are determined through parameter identification based on CFD simulation results. We calculated the aerodynamic forces under different modal deformation amplitudes and used the least squares method to fit the linear relationship between the generalized velocities and the corresponding changes in aerodynamic forces. $k_{D,i}$ quantifies the additional drag induced by modal shape variations, while $k_{L,i}$ represents the aerodynamic damping effect associated with modal velocities, which affects the lift force.

4. Although Fig. 3 defines several coordinate frames, the text remains difficult to follow. A more concise summary linking each frame directly to its modeling purpose (e.g., for force projection, for attitude computation, etc.) would improve readability.

**Response 4：**

We sincerely thank the reviewer for the suggestion regarding the clarity of the coordinate system definitions. Immediately after defining the coordinate systems, we have added a functional summary that explicitly associates each coordinate system with its specific modeling purpose. The related modifications have been highlighted in yellow in the manuscript.

5. The FSI section briefly describes the mesh and coupling scheme, but parameters such as solver coupling steps, convergence criteria, mesh update methods, and time-step strategies are not provided. These details are important for reproducibility.

**Response 5：**

We thank the editor for pointing out the lack of these technical details. We have supplemented the detailed FSI settings: a bidirectional implicit coupling strategy is employed to handle the interaction between the flexible parachute and the airflow. The time step is set to 0.01s, with a maximum of 30 coupling iterations performed per time step. To accommodate the large deformations of the parachute, a diffusion-based smoothing method is applied to update the fluid domain mesh, with the convergence criterion set to an RMS residual of 1e-4. The additions have been highlighted in yellow in the manuscript.

6. The manuscript claims ≤5% error between dynamics simulation and FSI results, but Table/quantitative comparison is missing. Adding a small table summarizing error metrics (horizontal displacement, vertical displacement, attitude angle) would substantiate the conclusion.

**Response 6：**

We thank the reviewer for the suggestion. We fully agree that quantitative comparative data are needed to support the conclusions on accuracy presented in the paper. In the revised manuscript, we have added Table 3. This table details the relative errors between the dynamic model proposed in this paper and the high-fidelity FSI simulation results in terms of horizontal displacement and vertical displacement. Comparative data indicate that the conclusion of ≤5% mentioned in the text holds true for the vast majority of working conditions.

7. The deviation analysis (Fig. 11) explains flow-induced deformation effects, but the argument would be clearer if supported by quantitative FSI outputs (pressure contours, deformation amplitude, aerodynamic center shift).

**Response 7：**

We thank the reviewer for the suggestion. In the revised manuscript, we have added quantitative descriptions of the deformation, highlighted in yellow. We compared the aerodynamic center positions and force coefficients between the rigid model and the FSI model. Compared to the rigid benchmark, flow-induced deformation causes the aerodynamic center to shift rearward by approximately 26%.

8. Add a short paragraph explaining how the Kane-based multibody model used here differs from, and is less/more physically enriched than, modern physics-embedded dynamic models such as those discussed in doi:10.1016/j.ress.2025.111262. This comparison will better position the contribution in the context of current literature.

**Response 8：**

We thank the reviewer for the suggestion. We have added a comparative analysis between the two, as highlighted in yellow. Modern physics-embedded methods effectively integrate physical laws with data-driven algorithms, showing great potential in computational efficiency and uncertainty quantification for complex systems. In contrast, the Kane-based multibody model developed in this paper serves as a high-fidelity, deterministic physical benchmark. Unlike methods that approximate dynamics by learning constraints, the Kane formulation explicitly constructs the equations of motion based on precise kinematic chains and vector mechanics. This characteristic gives it a unique advantage in analytically capturing the strong geometric nonlinearities inherent in the separation process.

9. Figures showing trajectories and flow fields (e.g., Figs. 8–13) require consistent axis labels, units, and clearer legends. Some curves are difficult to differentiate.

**Response 9：**

We thank the editor for the meticulous review of the figure quality. We have carefully revised Figures 8-13 to improve their clarity and consistency. We have unified the font style, size, and unit representation for all axis labels. To address the issue of difficulty in distinguishing between curves, we have adopted different line styles (solid and dashed lines) and a high-contrast color scheme. We attempted to implement the suggested unification of axis ranges across all subplots. However, due to the significant variation in the magnitude of changes among different state variables, forcing a uniform scale would obscure the details of the trajectories. Therefore, to ensure the readability of local variation details, independent axis ranges have been retained for some of the subplots.

10. For transparency, briefly mention limitations such as simplified aerodynamics, absence of wind disturbances, and uncertainties in structural parameters. Suggest future directions (e.g., stochastic wind fields, full-order flexible models, experimental validation).

**Response 10：**

We thank the reviewer for the valuable suggestion. We fully agree that an in-depth discussion of the study's limitations and a clear presentation of future prospects are crucial for enhancing the rigor and depth of the paper. In response to your comments, we have elaborated on the limitations of this study in an objective manner, and have proposed feasible future research directions accordingly. The relevant revisions are highlighted in yellow.
